# Molecular and Morphological Characteristics of a Novel Cyst Nematode in the Rhizosphere of *Artemisia lavandulaefolia* DC. in Gansu Province, Northwest China

**DOI:** 10.3390/pathogens13100881

**Published:** 2024-10-09

**Authors:** Wei Guo, Huixia Li, Xuejuan Wei, Ning Luo, Mingming Shi

**Affiliations:** Biocontrol Engineering Laboratory of Crop Disease and Pests of Gansu Province, College of Plant Protection, Gansu Agricultural University, Lanzhou 730070, China; guow@gsau.edu.cn (W.G.);

**Keywords:** *Artemisia lavandulaefolia* DC., *Cactodera chenopodiae*, molecular identification, morphological characteristics

## Abstract

Cyst nematodes are obligate parasitic nematodes found in the fields of many cultivated crops. These nematodes, which have great economic importance, pose a threat to food security, though they are frequently ignored or misdiagnosed as pests because of covert parasitism. A cyst nematode population parasitizing on *Artemisia lavandulaefolia* DC., one of the traditional Chinese medicines was collected in Gansu Province. The species was diagnosed using integrative taxonomy and molecular approaches. The cyst population is spherical or lemon-shaped, light brown or dark brown in color, with a long neck and a protruding vulval cone. The stylet of the second-stage juvenile is strong, and the front end of the ball at the base of the stylet is concave; the median bulb and excretory pore are prominent; the tail is blunt and circular, and the transparent tail is usually shorter than the stylet. A phylogenetic analysis was carried out using the internal transcribed spacer (ITS) and 28S genes of ribosomal DNA, which further confirmed the presence of *Cactodera chenopodiae*. According to our literature review, this is the first report on *C. chenopodiae* in Compositae. By following this research, we can better understand the challenges posed by *A. lavandulaefolia* DC. and develop effective strategies for managing its spread and impacts. This will help to protect vulnerable ecosystems and ensure the sustainability of agricultural and forestry activities in affected areas.

## 1. Introduction

*Artemisia lavandulaefolia* DC. is a traditional Chinese medicine widely distributed in China, belonging to the Compositae family. It has extremely high clinical value, with enormous development potential and abundant resources [1,2]. For example, previous studies have shown the possible molecular mechanism of sesquiterpene 5α-Hydroxycostic acid, the active ingredient of the traditional She medicine *A. lavandulaefolia* DC., in the treatment of rheumatoid arthritis [3]. However, biotic and abiotic constraints limit the production of Chinese medicinal herbs in China. Among the biotic constraints, nematodes have been reported to cause significant damage. Phyto-parasitic nematodes are often considered one of the most damaging pests among the factors restricting crop and Chinese medicinal herb cultivation globally [4,5,6].

The genus *Cactodera* is an important component of plant-parasitic cyst nematodes. The genus *Cactodera* was established by Krall and Krall [7]. Subbotin et al. [8] provided a key to the species of the genus *Cactodera*, which currently contains 19 species [9,10]. In recent years, there have been continuous new discoveries about the genus *Cactodera*. In 2018–2021, during nematological surveys in several regions of Mexico, *Cactodera solani*, *C. rosae*, *Cactodera* sp. (a new species), and *C. herba* sp. n. were recovered and distinguished from soil and root samples; among them, *C. herba* sp. n. was collected from liverseed grass [10]. In 2020, a *Cactodera* species was collected from the roots and surrounding soil of potato in Guizhou Province, which is herein described as a new species, *Cactodera guizhouensis* n. sp., based on a comparison of morphological and molecular characteristics [9]. These new discoveries have enriched the species of the genus *Cactodera*.

Due to intraspecific variation and the overlap of intraspecific morphological measurements, it is difficult to completely distinguish Heteroderinae species based on morphology, especially between closely related species [11,12]. The morphological identification method for species belonging to the genus *Cactodera* is mainly based on the L/W ratio, the fenestral diameter, and whether vulval denticles are present or absent in the cyst, as well as the length of the stylet, tail, and hyaline region in second-stage juveniles [8,11,13]. With the rapid development of molecular biology technology, the sequence of the ITS rRNA gene and molecular features such as the D2–D3 region of the 28S rRNA gene provide new methods for the accurate identification of Heteroderinae species [14,15,16,17]. Therefore, the comprehensive morphological characteristics and molecular biology methods of nematodes make their identification more accurate and are also considered standard detection methods [18].

Longnan is the area with the highest vegetation coverage and the highest number of biological species in Gansu. The rich vegetation types in this region can be roughly divided into deciduous broad-leaved forests, mixed coniferous and broad-leaved forests, subalpine coniferous forests, and alpine shrub meadows [19]. There are more than 4000 seed plants and 469 medicinal plants [20]. The rich and diverse plant species within the area provide possibilities for the parasitism of cyst nematodes. In addition, there is little research on the harm of cyst nematodes in the mountainous areas of Longnan, and only soybean cyst nematodes have been found to be harmful to soybeans [21]. Therefore, it is necessary to clarify the nematode species in this area to enrich their diversity and improve management.

In light of these observations, white females were identified at the roots of *A. lavandulaefolia* DC. which exhibited poor growth. Additionally, a population of cyst nematodes was collected and isolated from the rhizosphere soil in Longnan City, Gansu Province. The identification combined morphological features with the D2–D3 region of the 28S rRNA gene and ITS-rDNA gene sequences to determine the species and enrich the diversity of cyst nematodes in the region. Furthermore, it can also provide a scientific basis for the prevention and control of nematode diseases in traditional Chinese medicine.

## 2. Materials and Methods

### 2.1. Separation and Collection of Cysts

The simple floating separation method was used to isolate the rhizosphere cyst nematodes from *A. lavandulaefolia* DC. The cysts were picked under a stereomicroscope (Olympus, Tokyo, Japan) and stored at 4 °C for later use. At the same time, we observed whether the root system of *A. lavandulaefolia* DC. was parasitized by white females under a stereomicroscope.

### 2.2. Morphological Identification of Cyst Nematodes

The main morphological characteristics of the cysts, vulval cones, and second-stage juveniles were observed, measured, and photographed under optical microscopy (Zeiss, Jena, Germany) and stereomicroscopy (Olympus, Tokyo, Japan). The production method for cyst vulval cone slides is as follows: the plump cyst was cut open and trimmed on a glass slide filled with sterile water; the cyst, filled with impurities, was carefully removed with a small brush; and the trimmed vulval cones were then bleached in 40% hydrogen peroxide for 3 min, transferred to 70%, 95%, and 100% alcohol for dehydration, and finally transferred to clove oil for transparency. After achieving transparency, the trimmed vulval cones were moved to a glass slide for observation and photography. A second-stage juvenile slide was made using the experimental method of Xie et al. [22]: the second-stage juvenile was fixed after heat killing with TAF fixative, with water used as a floating carrier. The fixed nematodes were covered with a cover glass and sealed with a sealing agent. The morphology was observed, and the morphological characteristics were measured. The methods of Xie et al. [22] and Duan Yuxi [23] were used as references for morphological data analyses, and the method of Perry [24] was used to classify and describe the characteristics of the nematodes for species identification.

### 2.3. DNA Extraction from Cyst Nematodes

The method of Ni et al. [25] was used to extract DNA from individual plant nematodes. An individual linear nematode was washed with double-distilled water (ddH_2_O) and subsequently cut into smaller fragments. A single second-stage juvenile was randomly selected and washed three times with ddH_2_O. Then, it was transferred to a PCR tube containing 10 µL of nematode lysis solution. The mixture was centrifuged at 3000 r/min for 2 min, frozen quickly in liquid nitrogen for 1 min, and heated at 85 °C for 2 min; 1 mg/mL of protease K 1 µL was added, and then centrifuged again. The PCR instrument was incubated at 65 °C for 15 min to allow protease K to degrade proteins and release DNA. Then, protease K was inactivated at 95 °C for 10 min. Finally, the mixture was centrifuged at 14,000 r/min for 2 min, and the DNA supernatant was stored at −20 °C for later use.

### 2.4. PCR of ITS-rDNA and D2-D3 Region of 28S-rDNA Sequences in Cyst Nematodes

(1) PCR system. The PCR conducted in this experiment was carried out using a 25 µL reaction system, as shown in Table 1:

(2) PCR conditions. The ITS-rDNA region was amplified using universal primers TW81 (5′-GTTTCCGTAGGTGAACCTGC-3′) and AB28 (5′-ATATTGCTTAAGTTCAGCGGT-3′) [11]. The D2-D3 region of 28S-rDNA was amplified using universal primers D2A (5′-ACAAGTACCGGGGAAAGTTG-3′) and D3B (5′-TCGGAAGGACAGC-TACTA-3′) [9]. The PCR reaction conditions for the ITS-rDNA region and the D2-D3 region of 28S-rDNA were consistent, as shown in Table 2:

### 2.5. Cloning and Sequencing of PCR Products

The method of Wang Zhikun [26] was referenced and improved. The amplified products were detected by 2% agarose gel electrophoresis, and then recovered using a DNA gel recovery kit. After connecting the recovered products with the pMD19-TVector carrier, they were transformed into Escherichia coli DH5 α competent cells. The cells were incubated in an ice bath for 30 min, followed by a 42 °C metal bath for 45 s, then placed on ice for 1 min before adding 500 µL of LB liquid culture medium. After shaking at 37 °C and 180 r/min for 60 min, 100 µL was uniformly coated onto the LB solid culture medium and left overnight in a 37 °C incubator. A certain number of discrete monoclonal clones were collected using a 10 µL sterile gun tip, placed in a 10 mL (LB) liquid culture centrifuge tube containing 60 µg/mL Amp, and put in a shaking table at 37 °C and 180 r/min for 14 h. PCR was performed and, finally, the PCR products were sent to Xi’an Sangon Biotechnology Company for sequencing.

### 2.6. Sequence Phylogenetic Analysis of PCR Products

The sequences of closely related species were obtained by downloading them from GenBank. The sequences were aligned using MAFFT v7.149b, and the software Gblock 0.91b was used to select conserved regions of the sequences. The Bayesian method was used for phylogenetic analysis, the optimal model in MrBayes 3.2.6 was used to construct a phylogenetic tree, and FigTree V.1.4.3 was used to correct the phylogenetic tree [27]. A phylogenetic tree constructed based on ITS-rDNA and the D2-D3 region of 28S-rDNA was selected as an outlier based on existing reports.

## 3. Results

### 3.1. Morphology of Rhizosphere Cyst Nematode of Artemisia lavandulaefolia DC.

**Figure 1:**
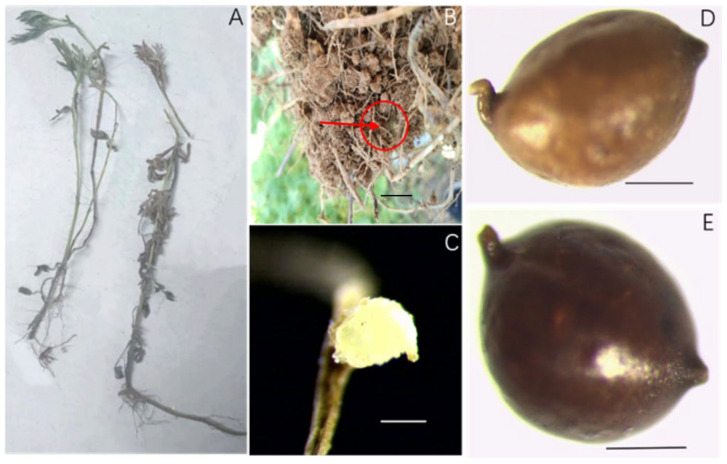
Light micrographs of the rhizosphere cyst of *Artemisia lavandulaefolia* DC. (**A**). *A. lavandulaefolia* DC.; (**B**,**C**). Females on *A. lavandulaefolia* DC.; (**D**,**E**). Cyst (Scale bar: (**B**) = 1 cm; (**C**–**E**) = 200 µm).

The morphology of the rhizosphere cyst nematode of *A. lavandulaefolia* DC. is shown in Figure 1. Figure 1A shows *A. lavandulaefolia* DC., with the white female parasitizing the roots of *A. lavandulaefolia* DC. (Figure 1B,C). The surface of the white female has a sub-crystalline layer (Figure 1C). The cyst population is spherical or lemon-shaped, light brown or dark brown in color, with a long neck and a protruding vulval cone (Figure 1D,E).

### 3.2. Morphological Identification of Cyst Population

**Figure 2:**
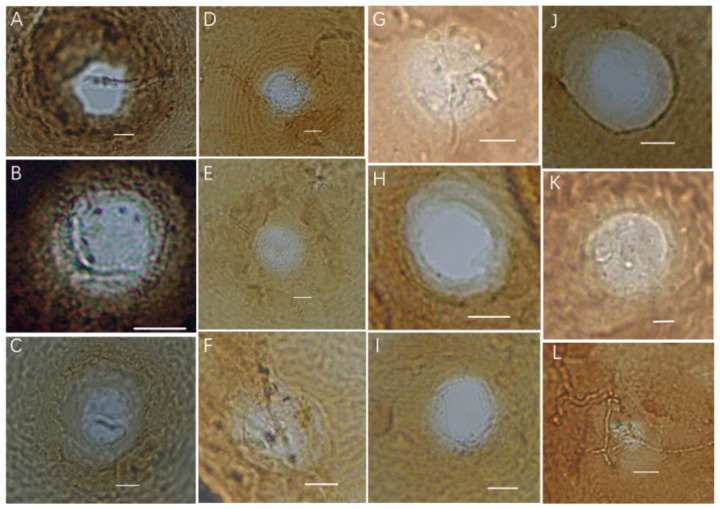
Light micrographs of vulval cones of the cyst population. (**A**–**G**,**L**). Circumfenestrate (with underbridge); (**H**–**K**). Circumfenestrate (without underbridge) (Scale bar: (**A**–**J**) = 10 μm; (**K**) = 5 μm; (**L**) = 20 μm).

**Figure 3:**
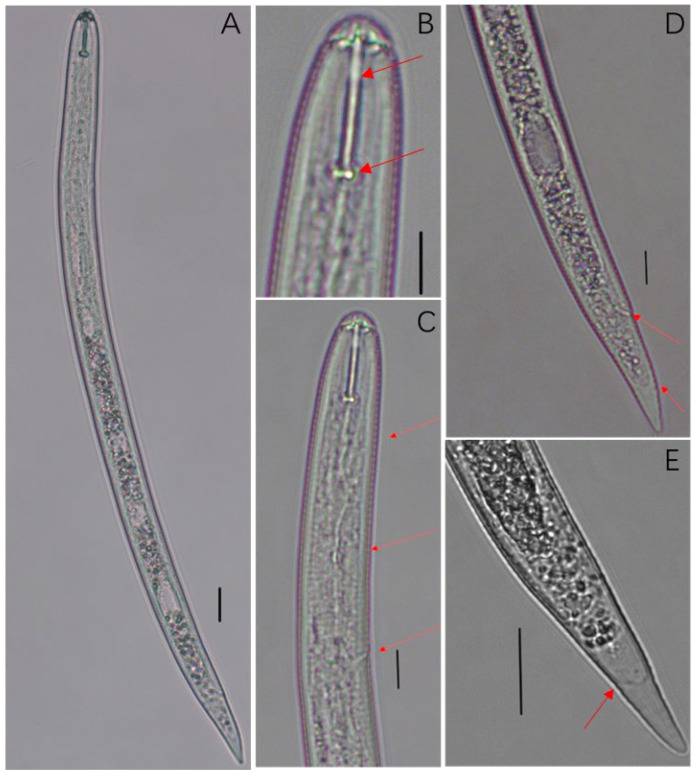
Light micrographs of second-stage juvenile of the population. (**A**). Entire body; (**B**). Stylet; (**C**). Anterior region of second-stage juvenile; (**D**). Tail region of second-stage juvenile; (**E**). Tail (Scale bar: (**A**) = 20 μm; (**B**–**D**) = 10 μm; (**E**) = 20 μm).

The micrograph of the vulval cones of the population of *A. lavandulaefolia* DC. cyst nematodes is shown in Figure 2, and the micrograph of the second-stage juveniles of the cyst population is shown in Figure 3. The morphological identification results are as follows: all photos show that the vulval cones are circumfenestrate (Figure 2A–L); some photos can show an underbridge (Figure 2A–G,L) and some do not show an underbridge (Figure 2H–K).

The second-stage juveniles are worm-like, and after heat killing, the worm-like body slightly bends towards the abdomen (Figure 3A). The head is significantly constricted, the stylet is strong, and the front end of the ball at the base of the stylet is concave (Figure 3B,C). The median bulb and excretory pore are prominent (Figure 3B). The tail is blunt and circular, with a transition from the tail to the transparent tail area forming a U-shaped or V-shaped contour. The transparent tail is usually shorter than the stylet (Figure 3D,E). The morphological description of the cyst population is consistent with the morphology of the parasitic Chenopodiaceae plant cyst nematode reported by Feng et al. [28], and the cyst population was preliminarily identified as *Cactodera chenopodiae*.

We compared and analyzed the morphological measurements of this population with those of the Liaoning population of *C. chenopodiae*. As can be seen in Table 3, the measurement range of the Longnan population and the Liaoning population described by Feng et al. [28] basically overlap, but there are differences in some measurement values. The main manifestations of this study population are as follows: cyst length [553.10 ± 15.20 (355.71–738.58) μm vs. (423.4–585.4) μm], cyst width [411.05 ± 13.42 (277.89–609.15) μm vs. (283.0–398.1) μm], a [21.84 ± 0.29 (18.67–26.40) μm vs. (18.0–24.7) μm], c [12.83 ± 0.30 (10.26–17.49) μm vs. (9.8–12.6) μm], EP [108.60 ± 0.95 (95.68–124.88) μm vs. (103.9–121.3) μm], which are slightly larger than those of the Liaoning population; the body length of the second-stage juvenile (J2) [485.24 ± 4.55 (397.64–559.73) μm vs. 423.4–585.4 μm], the stylet [23.29 ± 0.21 (19.35–25.90) μm vs. (21.9–25.9) μm], and the tail length [38.47 ± 0.80 (27.69–47.62) μm vs. (39.1–50.6) μm] are slightly smaller than those of the Liaoning population.

### 3.3. Molecular Phylogenetic Analysis of Cyst Population

This study obtained a total of four D2–D3 region of 28S rDNA sequences from cyst populations, with a length of 577 bp and GenBank accession numbers of OQ625739, OQ626201, OQ626167, and OQ626168, respectively. Four ITS rDNA region sequences, with a length of 906 bp and GenBank accession numbers OQ625810, OQ625811, OQ625812, and OQ625813, were also obtained.

**Figure 4:**
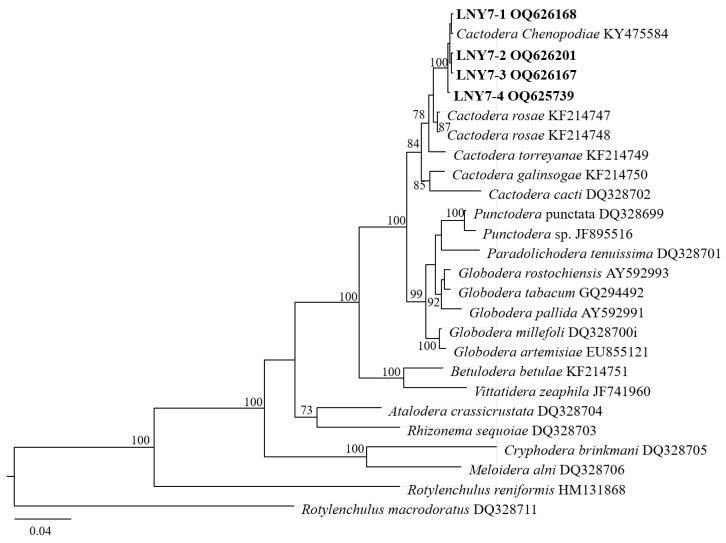
Molecular phylogenetic tree of cyst population 1 (highlighted in bold) inferred from the D2–D3 region of 28S-rDNA.

In the D2–D3 region of the 28S rDNA phylogenetic tree (Figure 4), the four cyst populations in this study clustered with the one cyst population in the gene library of Liaoning *C. chenopodiae* to form independent evolutionary branches, with a confidence level of 100%. The BLAST alignment results of the D2–D3 region of the 28S rDNA showed that the sequence similarity between the four spore sac populations in this study and this nematode population (KY475583) was the highest, at 100%.

**Figure 5:**
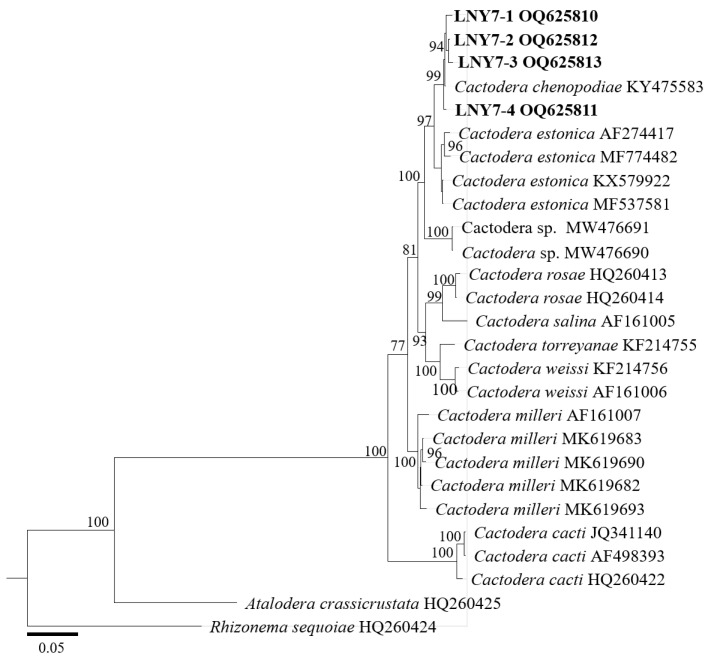
Molecular phylogenetic tree of cyst population 1 (highlighted in bold) inferred from the ITS-rDNA region.

In the phylogenetic tree of the ITS rDNA region (Figure 5), the four cyst populations in this study clustered with one cyst from the gene pool of Liaoning *C. chenopodiae* to form an independent evolutionary branch, with a confidence level of 99%. The BLAST alignment results of the ITS rDNA region showed that the sequence similarity between the four spore sac populations in this study and this nematode population (KY475583) was high, at 100%.

Based on the analysis of morphological and molecular biological characteristics, the population of cysts isolated from the rhizosphere of *A. lavandulaefolia* DC. in Longnan was identified as *C. chenopodiae*.

## 4. Discussion

*Cactodera chenopodiae* belongs to the order Tylenchida, family Heteroderidae, subfamily Heteroderinae, and genus *Cactodera*. The genus *Cactodera* has significant economic importance worldwide, and currently, 19 effective species of this genus have been reported [9,10,27,28,29,30]. In China, four species of this genus have been reported, namely *C. thornei*, *C. cacti*, *C. chenopodiae*, and *C. tianzhuensis*. This study investigated the formation of females from the root system of *A. lavandulaefolia* DC., indicating that *C. chenopodiae* can infect *A. lavandulaefolia* DC. and complete its life cycle. In China, only Feng et al. [28] found this cyst nematode parasitic on Chenopodiaceae plants. In Gansu Province, this genus has only been reported in the Tianzhu area [9] and has not been reported in other regions yet.

Among the effective species of the genus *Cactodera*, 12 species have been found to have males; however, 4 species of the genus *Cactodera—C. estonica*, *C. radicale*, *C. rosae*, *C. tianzhuensis*—lack males [9,28,29]. These species can be distinguished by different morphological characteristics. For example, *C. tianzhuensis* n. sp. can be distinguished from *C. acnidae* by its shorter fenestral diam, longer J2 body length, and longer tail length. *C. tianzhuensis* n. sp. is differs from *C. estonica* by having a smaller L/W cyst ratio, as well as a longer J2 body length, tail length, and hyaline tail length [11]. *C. chenopodiae* can also be distinguished from other species based on its morphological characteristics, and coincidentally, no males were found in the *C. chenopodiae* population parasitic on *A. lavandulaefolia* DC. in this study, which is consistent with the results of Feng et al. [28]. This indicates that the cyst nematode may have parthenogenesis or hermaphroditism, and its specific reproductive mode still needs further research. The morphological characteristics of the *C. chenopodiae* population in this study are basically consistent with those of the Liaoning population [28], but some measurement values have slight differences (detailed information is listed in Table 3); it is speculated that this difference may be related to factors such as the ecological environment, host nutrition, or climate of these two cyst populations [31].

The correct identification of nematode species is the basis for preventing and controlling nematode diseases. Different species can be preliminarily distinguished by morphological differences, but some morphological characteristics of nematodes may overlap between different populations, making it difficult to accurately identify them at the species level and genotype. Sensitive molecular detection methods do not rely on environmental factors, making the identification of plant-parasitic nematodes particularly important [32]. This study conducted a molecular biology analysis on the ribosomal ITS-DNA region and D2–D3 region of the 28S rDNA gene sequences of the *A. lavandulaefolia* DC. cyst nematode population in Longnan. The results showed that the cyst populations of *A. lavandulaefolia* DC. in Longnan and *C. chenopodiae* in Liaoning were clustered into an independent lineage. This study further confirmed that the cyst population of *A. lavandulaefolia* DC. in Longnan was *C. chenopodiae* using molecular biology methods. By utilizing morphological characteristics and molecular biology methods, different species of nematodes can be well distinguished [33,34,35,36]. In addition, some rapid and reliable methods have also been used for the identification of plant-parasitic nematodes [37,38]. In the future process of nematode identification, attention should be paid to the development and utilization of accurate and rapid methods for different nematode species, which will contribute to the development of nematode taxonomy.

This research highlights a significant finding in the field of nematode research, specifically concerning the cyst nematode species *C. chenopodiae*. Prior to this study, this nematode had only been detected in Liaoning Province, China [28], and its known host range did not include plants belonging to the Compositae family. However, the research described here reveals that *C. chenopodiae* can also infect and cause harm to Compositae plants, thereby expanding the known host range of this nematode genus. This discovery is noteworthy, as it underscores the need for further investigation into the geographical distribution and host range of *C. chenopodiae*.

Understanding the full extent of the nematode’s reach and the range of plant species it can infect is crucial for developing effective management strategies to mitigate its potential negative impacts on agriculture and natural ecosystems. There are currently few research reports on the control of *C. chenopodiae*; this study has identified a new host plant for *C. chenopodiae*, which plays an indispensable role in using RNA interference and other methods for the prevention and management of *C. chenopodiae* to ensure the safe production of valuable host plants such as *A. lavandulaefolia* DC. By expanding our knowledge of the nematode’s biology and ecology, researchers can better assess the risks associated with its presence and devise targeted control measures to protect vulnerable plant species.

## Figures and Tables

**Table 1:** DNA sequencing PCR reaction system.

Reaction Material	Concentration	Volume (µL)
Template DNA	-	2
Upstream primer	10 μmol/L	1
Downstream primer	10 μmol/L	1
PCR Taq Mix	-	12.5
ddH_2_O	-	8.5
Total volume	-	25

**Table 2:** DNA sequencing PCR system.

No. of Cycles	Temperature/°C	Time	Step
1	95	4 min	Pre-denaturation
34	95	30 s	Denaturation
	56	30 s	Annealing
	72	1 min	Extension
1	72	10 min	Extension

**Table 3:** Morphometric comparison of the Longnan population with the reported population.

Morphological Characters	Longnan Population	Liaoning Population [28]
Cyst number	40	20
Cyst length	553.10 ± 15.20 (355.71–738.58)	423.4–585.4
Cyst width	411.05 ± 13.42 (277.89–609.15)	283.0–398.1
L/W	1.36 ± 0.02 (1.11–1.65)	-
Fenestral length	25.39 ± 0.56 (18.75–37.55)	19.9–26.3
Fenestral width	22.46 ± 0.51 (15.58–30.04)	-
J2 number	40	20
Body length	485.24 ± 4.55 (397.64–559.73)	423.4–585.4
Body width at mid-body	22.29 ± 0.20 (20.33–24.95)	283.0–398.1
a	21.84 ± 0.29 (18.67–26.40)	18.0–24.7
c	12.83 ± 0.30 (10.26–17.49)	9.8–12.6
c′	3.34 ± 0.08 (2.04–4.37)	2.7–4.0
Lip region height	4.44 ± 0.12 (3.14–6.12)	-
Lip region diam	9.06 ± 0.10 (6.96–10.24)	-
St	23.29 ± 0.21 (19.35–25.90)	21.9–25.9
MB	79.84 ± 0.81 (67.95–94.13)	-
DGO	3.82 ± 0.06 (3.00–4.31)	-
EP	108.60 ± 0.95 (95.68–124.88)	103.9–121.3
MBW	10.68 ± 0.17 (8.41–13.33)	-
ABW	11.66 ± 0.26 (8.50–13.96)	-
Tail	38.47 ± 0.80 (27.69–47.62)	39.1–50.6
Hyaline portion tail	18.59 ± 0.33 (14.87–24.22)	14.87–24.22

The data in the table are expressed as mean ± SD; the data in parentheses are range values. unit µm.

## Data Availability

Data are contained within the article.
